# A Case of Male Osteoporosis: A 37-Year-Old Man with Multiple Vertebral Compression Fractures

**DOI:** 10.1155/2017/6328524

**Published:** 2017-07-16

**Authors:** Suhaib Radi, Andrew C. Karaplis

**Affiliations:** Division of Endocrinology, Department of Medicine, Jewish General Hospital, McGill University, Montreal, QC, Canada H3T 1E2

## Abstract

While the contributing role of testosterone to bone health is rather modest compared to other factors such as estradiol levels, male hypogonadism is associated with low bone mass and fragility fractures. Along with stimulating physical puberty by achieving virilization and a normal muscle mass and improving psychosocial wellbeing, the goals of testosterone replacement therapy in male hypogonadism also include attainment of age-specific bone mineral density. We report on a 37-year-old man who presented with multiple vertebral compression fractures several years following termination of testosterone replacement therapy for presumed constitutional delay in growth and puberty. Here, we discuss the management of congenital hypogonadotropic hypogonadism with hyposmia (Kallmann syndrome), with which the patient was ultimately diagnosed, the role of androgens in the acquisition of bone mass during puberty and its maintenance thereafter, and outline specific management strategies for patients with hypogonadism and high risk for fragility fractures.

## 1. Introduction

Osteoporosis in men is a serious disease that is underdiagnosed, undertreated, and often complicated by fragility fractures and their associated morbidity and mortality [1, 2]. Worldwide, nearly forty percent of all osteoporotic fractures in individuals over the age of 50 occur in men [3]. The mortality rate following a hip fracture in men is 37% in the first year, higher than that observed in women [4]. Male osteoporosis is often secondary, with the most common causes being corticosteroid use, excessive alcohol intake, and hypogonadism, including androgen deprivation therapy for prostate cancer [4]. To increase awareness of this disorder and the characteristics of its presentation, we report the case of a 37-year-old man presenting with multiple vertebral compression fractures.

## 2. Case Report

A 37-year-old man presented to the emergency department at our hospital on October 2012 with a 4-month history of worsening lower back pain. There was no history of trauma or falls. He was unemployed and lived with his mother. He took no medications or supplements, did not smoke, consume excessive alcohol, or use illegal drugs, and had never been treated with corticosteroids. He had no history of prior fractures. Family history was negative for gonadal, endocrine, or bone diseases. Review of systems was remarkable for markedly delayed puberty for which he had received testosterone injections starting at 16 years of age. Three years later, testosterone replacement therapy was terminated by the treating physician, after he responded well in terms of growth and development of secondary male sexual characteristics. He remarked decreased libido for several years and absence of morning erections.

On examination, he was a well-nourished gentleman in obvious distress due to pain at the level of the lower thoracic/lumbar spine. He had an eunuchoidal body habitus with the arm span (181 cm) exceeding the height (174 cm). The pupils were round, equal, and reactive to light. Visual fields were normal to confrontation. The skin turgor was normal, the mucous membranes were moist and the teeth were in good condition with no decay. Examination of the head and neck was remarkable for hyposmia to a number of odorants. The cardiovascular, respiratory, and abdominal exams were within normal limits. There was no edema in the extremities and neurological assessment was normal. Examination of the integument was remarkable for sparsity of pubic hair. The penile length was normal but testicular volume was decreased (approximately 5 cm^3^ as determined using the Prader orchidometer).

On biochemistry, serum levels for luteinizing hormone (LH), follicle-stimulating hormone (FSH), testosterone, and estradiol were extremely low while baseline values for serum creatinine, electrolytes, thyroid function tests [thyroid-stimulating hormone (TSH) and free T4], prolactin, cortisol, parathyroid hormone (PTH), and alkaline phosphatase activity were all within normal limits. Osteocalcin levels were increased while serum 25(OH) vitamin D levels were insufficient (Table 1). Complete blood count showed a normochromic normocytic anemia. Serum protein electrophoresis was unremarkable.

**Figure 1:**
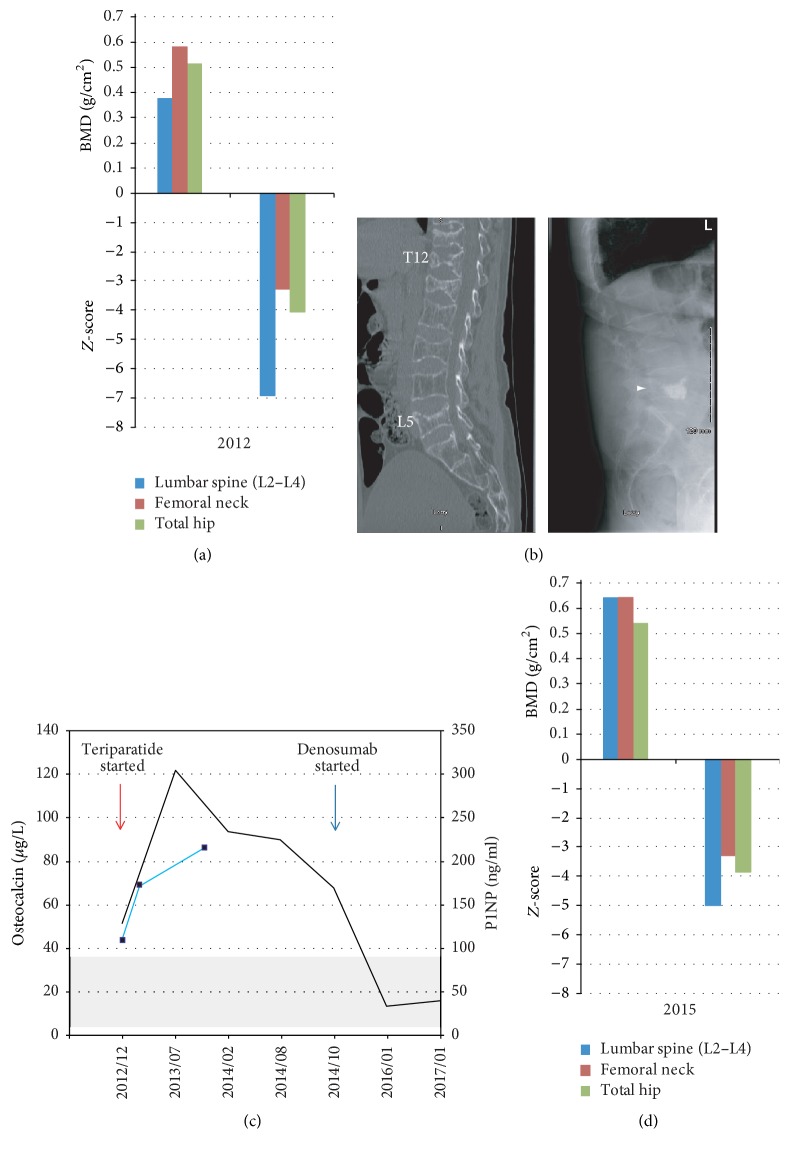
(a) BMD measurements and *Z*-scores at lumbar spine, femoral neck, and total hip at initial presentation. (b) Left: sagittal view of spine CT scan showing compression fractures of T12, L1, L3, and L5, with diffuse osteopenia. Right: lateral spine X-ray showing cement in the body of L5 following vertebroplasty (arrowhead) and severe wedging of L1 and L3. (c) Changes in serum levels of osteocalcin (—) and P1NP (■—■) during the course of treatment. Shaded area represents normal reference values. (d) BMD measurements and *Z*-scores after completing two years of treatment with teriparatide followed by one year of denosumab.

Radiographic examination of the spine disclosed diffuse osteopenia with moderate wedging of several mid-thoracic vertebrae along with severe compression fractures of T11, T12, and L1 (80%–90% vertebral height loss). There was a 10% superior endplate collapse at L5. Dual-energy X-ray absorptiometry (DXA) scan of the skeleton reported a *Z*-score of −6.9 at the lumbar vertebrae (L2–L4), −3.3 at the femoral neck, and −4.1 at the total hip (Figure 1(a)).

Magnetic resonance imaging (MRI) of the brain with particular attention to the hypophysis was carried out. The pituitary gland was normal except for a 2.4 × 2.6 mm left anterior pituitary cyst with no evidence of a mass effect.

Based on the aforementioned history, physical examination, radiographic evaluation, and biochemical data indicative of hypogonadotropic hypogonadism with hyposmia, the patient was diagnosed with Kallmann syndrome with severe osteoporosis due to hypogonadism. He was given analgesia and was started on calcium (500 mg/day), vitamin D (2000 IU/day), and testosterone replacement therapy (Androgel™ 1% 2.5 g daily) while awaiting for government approval for teriparatide (Forteo™) use.

Three weeks later, he presented again to the emergency department with a 4-day history of being unable to ambulate due to severe low back pain following a fall from standing height. A CT scan of the spine disclosed a new L3 compression fracture, with 75% loss of height, along with an interval progression of the L5 superior endplate collapse (from 10% to 40% loss of height). The T11-L1 fractures remained unchanged (Figure 1(b), left panel). Given that the area of maximal pain was at the L5 level, the patient underwent L5 vertebroplasty with good analgesic response and near complete ambulatory recovery (Figure 1(b), right panel).

While convalescing, his application for teriparatide use was approved and treatment was initiated. Skeletal response to the drug was monitored using osteocalcin and procollagen type 1 N-terminal propeptide (P1NP) serum levels as surrogate markers of bone formation (Figure 1(c)). Concurrently, the dose of Androgel was gradually increased to 10 g daily. While on this dose, serum testosterone levels normalized and the anemia resolved. On December 2014, after completing 24 months of teriparatide treatment, he was switched over to sequential treatment with denosumab (Prolia™) 60 mg s/c q6 months. A repeat DXA scan performed on May 2015 (following two doses of denosumab) showed improvement of the bone mineral density at the lumbosacral spine, femoral neck, and total hip [*Z* score of −5 (from −6.9), −3.3 (from −3.3), and −3.9 (from −4.1)] (Figure 1(d)). He has been doing well since then, and while continuing with calcium, vitamin D, testosterone, and denosumab therapy, he has remained pain-free and has not sustained further fractures.

## 3. Discussion

Hypogonadism is a common cause of osteoporosis in men (rates ranging from 16 to 30% as the attributable cause) [reviewed in [5]]. It is classified either as hypergonadotropic (primary gonadal failure) or hypogonadotropic (secondary to a defect in the hypothalamic-pituitary axis) hypogonadism [6, 7], with the latter being due to congenital (defects in gonadotropin releasing hormone (GnRH) neurons, GnRH regulating neurons, and LH and FSH secreting cells) or acquired etiologies (structural defects or reversible causes). Congenital hypogonadotropic hypogonadism is subclassified into normosmic, where the sense of smell is intact (40%), or anosmic/hyposmic (absent/decreased sense of smell), the latter also being referred to as Kallmann syndrome (60%).

Kallmann syndrome is a rare developmental disorder, more prevalent in men (1 : 10,000 compared to 1 : 50,000 in women) [7]. It arises as a consequence of failed migration of gonadotropin-releasing hormone (GnRH) and olfactory neurons to the forebrain during intrauterine development. Clinically characterized by failure to initiate puberty due to insufficient gonadotropin release, it results in failure to develop secondary sexual characteristics and a mature reproductive system. Although the disease is often named after the German-American psychiatrist Dr. Kallmann who described its genetic aspects in 1944 [8], it was first described in 1856 by Maestre de San Juan in a man with absent pubic hair, shrunken testes, decreased sense of smell, and aplasia of the olfactory bulbs at autopsy. Kallmann syndrome is mainly sporadic but can be familial. Multiple genetic mutations have been reported to underlie its pathogenesis, the most common one being* KAL1*, encoding the extracellular glycoprotein anosmin-1, that is responsible for the X chromosome-linked recessive form of the disease. Mutations in a number of other genes have also been reported although all together they may account for less than 30% of these cases. Kallmann syndrome is usually a clinical diagnosis defined by the presence of idiopathic hypogonadotropic hypogonadism and anosmia or hyposmia. Additional abnormalities that may aid in the early diagnosis of the disease including synkinesia* (KAL1)*, dental agenesis and bony anomalies (*FGF8/FGFR1* or* KAL2*), hearing loss* (CHD7, SOX10)*, renal agenesis, and cleft lip and palate [reviewed in [9]]. A number MRI findings have been reported in patients with Kallmann syndrome, including absent or hypoplastic olfactory bulb and sulci in addition to hypoplastic anterior pituitary [10]. Although these findings may be useful if the clinical picture is not clear, they are not necessary for the diagnosis.

Androgens along with estrogens support the acquisition of bone mass during puberty and its maintenance thereafter [extensively reviewed in [11]]. In men, 15% of estrogen is secreted directly from the testes, and the remaining 85% is derived from peripheral conversion of testosterone by the aromatase (CYP19A1) enzyme [12]. Testosterone on the other hand is made by the Leydig cells of the testicles and acts unmodified or following conversion to the more potent dihydrotestosterone (DHT). The circulating estrogen and androgen levels are controlled by the gonadotropins FSH and LH via the hypothalamic-pituitary-gonadal axis. However, only 1–5% of circulating sex hormones is biologically active, comprising the free fraction not bound to sex hormone-binding globulin (SHBG), albumin, and other proteins.

The effects of androgens on bone are exerted upon binding with high affinity to the androgen receptor (AR; NR3C4) [13]. The androgen receptor has been identified in osteoblasts (bone cells responsible for the deposition of new bone matrix and its mineralization), osteocytes (osteoblasts entombed within the mineralized matrix and interconnected by processes via a canalicular system that extends all the way to the surface of bone used to sense and respond to changes in mechanical forces) and their progenitors, the pluripotent mesenchymal bone stromal cells, and osteoclasts (bone cells that resorb the mineralized matrix arising from hematopoietic precursors).

In both sexes, prepubertal growth velocity is greater in the appendicular skeleton (comprising long bones like the femur and radius) than in the axial (skull, spine, sternum, and the ribs) skeleton. Therefore, patients like ours with disorders of puberty exhibit so-called eunuchoid proportions, characterized by long limbs (arm span) relative to the spine (height).

The role of androgens on male bone metabolism is twofold. First, androgens stimulate bone formation during puberty. Second, androgens prevent bone resorption during and after puberty [14]. During growth, bone is shaped by modeling, a process that ensures the acquisition of the appropriate bone morphology and shapes skeletal elements and mass. Periosteal bone apposition is, for the most part, responsible for the enlargement of bones during growth. The periosteum is a thin layer of connective tissue that covers the external surfaces of most bones and is rich in osteogenic cells. Greater periosteal expansion during puberty accounts for larger bones and hence increased strength and reduced fracture risk. Interestingly, mouse models with targeted AR deletion at different stages of the differentiation program of the osteoblast lineage demonstrate no effect on cortical bone mass while those with global deletion of AR do show a delay in cortical bone mass accrual during puberty. Therefore, androgens do not exert their effects on cortical bone directly through actions on osteoblasts and their progenitors but rather indirectly via actions on some other cell type(s) or tissue(s), yet to be identified [15]. In contrast, the effects of androgens on cancellous bone are direct actions in cells of the osteoblast lineage. AR signaling in osteoblasts leads to a decrease in osteoclast numbers and bone resorption and is responsible for the protective effects of androgens on cancellous bone mass [16]. Finally, genetic evidence from mice with osteoclast-specific AR deletion indicates that androgen signaling in osteoclasts plays no role in the antiresorptive effect of androgens on the cancellous or cortical bone compartments [17].

In addition to bone development and growth, androgens are known to affect the maintenance of bone in men [11, 14]. The maintenance of skeletal mass in adulthood depends on the balance between bone resorption and formation during the continuous regeneration of the skeleton by the process of remodeling. Here, mature bone tissue is removed from the skeleton by resorption and new bone tissue is formed by ossification. Under physiological conditions, bone resorption and formation during remodeling are linked in time and space, being referred to as coupling. Androgens help to maintain bone mass during adult life by slowing the rate of bone remodeling and preserving the balance between resorption and formation, thus explaining the reduction in bone mass at all sites in our patient. In elderly and young hypogonadal men, loss of androgens contributes to the development of low BMD and bone fragility (osteoporosis), a prevalent and impactive metabolic disease.

It is also important to point out that, in men, in addition to androgens, a threshold level of bioavailable estradiol is needed to prevent bone loss, as it has a more dominant role than testosterone [12, 18]. Support for the concept of estrogen being more of a determinant on bone health than testosterone comes from “experiments of nature,” that is, from male patients with either estrogen resistance caused by mutations in the estrogen receptor gene [19] or aromatase deficiency, the enzyme responsible for aromatization of androgens into estrogen in extragonadal sites, including fat, brain, skin, endothelium, and bone [20]. The latter group of patients exhibited reduced bone mass at all sites and unfused epiphyses, with normal to high testosterone levels. Interestingly, restoration of bone mass and epiphyseal closure were reported following estrogen replacement, underlining the importance of estrogens in these processes [21]. Finally, selective estrogen receptor modulators (SERMs) which stimulate estrogenic action in bone can block bone loss in orchiectomy-induced, testosterone-depleted male mice [22].

Male patients treated for androgen deficiency can initiate replacement doses of testosterone if fertility is not desired and when there are contraindications [reviewed in [23]]. Testosterone therapy in Kallmann syndrome will achieve full virilization but will not achieve normal testicular volume nor fertility. If fertility is desired, it will need to be replaced with either combined FSH and human chorionic gonadotropin (hCG) or GnRH pump therapy [24]. Testosterone therapy of young, hypogonadal men such as our patient is associated with improvements in overall sexual activity scores, hair growth in several androgen-sensitive areas, fat-free mass, and muscle strength [23]. Although testosterone therapy in healthy, hypogonadal men increases bone mineral density [25, 26], the effects of testosterone on fracture risk are unknown. Different forms of testosterone application are available, such as intramuscular injections, oral, patch/gel/liquid for topical application, or intranasal application. These modalities are all equally effective in normalizing serum testosterone levels and the choice is usually based on patient's preference and cost [23]. However, appropriate dosing should be instituted. Based on the Endocrine Society Clinical Practice Guidelines, the starting dose for Androgel is 5–10 grams daily. In our patient, we opted to initiate treatment with a lower initial dose (2.5 g/day) and increase it progressively, given the long-term absence to testosterone exposure.

In our patient, testosterone replacement therapy had been terminated at the age of 19, after he responded well in terms of growth and development of secondary male sexual characteristics. Conflicting data has been reported about reversibility of hypogonadism in Kallmann syndrome. Some have reported reversibility, even after prolonged discontinuation of therapy [27], while others showed recurrence of the disease [28]. In the report by Raivio et al., sustained reversal of idiopathic hypogonadotropic hypogonadism was achieved in 10% after a mean treatment duration of 6 weeks [29]. The authors concluded that it is reasonable to do a treatment holiday for those patients. However, if a trial of therapy is to be instituted, continuous surveillance should be performed so as to detect and treat any recurrence [28, 30].

Approved treatments for men with low bone mass and high risk for fractures, including those due to hypogonadism, are bisphosphonates (alendronate, risedronate, and zoledronic acid), teriparatide, a recombinant form of human parathyroid hormone amino acids 1–34 [4], and denosumab [31], based on the findings of the ADAMO trial [32]. In addition, optimal calcium and vitamin D intake should be encouraged and specific life style changes be instituted, as needed. For those with hypogonadism and high risk of fractures, treatment should be added to testosterone therapy, whereas, in patients at medium risk of fracture, testosterone alone is usually sufficient. Our patient was treated with teriparatide for 24 months followed by denosumab, in addition to being on testosterone. Patients treated with teriparatide, when switched to denosumab, continue to show increase in bone mineral density, as reported in the DATA-Switch study [33]. Conversely, in the absence of consolidation therapy with denosumab, the large teriparatide-induced gains in BMD are abruptly lost [34]. Novel bone anabolic agents such as romosozumab or abaloparatide could be considered as alternatives to teriparatide in the near future. Osteocalcin and P1NP are bone turnover markers that can assist in monitoring the response to therapy. With bone anabolic agents like teriparatide, a rise in serum osteocalcin and P1NP levels is expected during the first 6 months of treatment, while serum levels decrease with antiresorptive agents, such as bisphosphonates and denosumab, suggestive of a satisfactory response to treatment [33, 35]. The anticipated bone turnover marker response was observed in our patient, as shown in Figure 1(c). Indeed, there was a concomitant improvement in bone mineral density measurements and he has not sustained additional fractures while continuing therapy.

Finally, it is well recognized that low testosterone causes anemia through decreased erythropoiesis, even in the absence of chronic disease, that is corrected following testosterone replacement therapy [36, 37]. A recent randomised trial in older men with low testosterone and anemia showed that testosterone treatment significantly increased hemoglobin levels in men with unexplained anemia and in those with anemia from known causes [38]. This was also demonstrated here, as the patient's normochromic normocytic anemia corrected completely after serum-free testosterone levels normalized. In addition to the known effect of testosterone in increasing erythropoietin concentrations which stimulate erythropoiesis, testosterone treatment is associated with the suppression of hepcidin and an increase in the expression of ferroportin and transferrin receptor [39]. These changes would potentially facilitate a greater bioavailability of iron from the storage/absorption sites, an increase in iron transport in the circulation, and the uptake at iron utilization sites, thereby further facilitating erythropoiesis. The effect of testosterone on erythropoiesis is dose-dependent and polycythaemia in response to overreplacement with testosterone is well-known [40].

In conclusion, Kallmann syndrome is an uncommon condition that should be suspected in patients with hypogonadotropic hypogonadism irrespective of age, and special attention should be paid to the clinical history for the presence of anosmia/hyposmia. It is a disease that is easily missed but at the same time readily treated with testosterone replacement therapy. Associated osteoporosis is quite common, and when associated with fragility fractures, it is best treated with bone anabolic agents followed by antiresorptive agents, specifically denosumab, in addition to testosterone. It is also important not to stop testosterone therapy without close surveillance as the rate of recurrence is high. Primary care physicians and specialists alike need to maintain awareness of male osteoporosis so that patients who are at risk for fractures are assessed and treated appropriately and in timely fashion.

## Figures and Tables

**Table 1:** Serum biochemistry.

Test	Result	Normal range
Creatinine	54	55–110 *µ*mol/L
Calcium	2.27	2.12–2.62 mmol/L
Phosphorus	1.31	0.70–1.45 mmol/L
Magnesium	0.76	0.70–1.23 mmol/L
Parathyroid hormone (PTH)	29	10–70 ng/L
Alkaline phosphatase	96	40–125 U/L
Osteocalcin	52.3	5–35 mg/L
25(OH) vitamin D	73	>75 nmol/L
Thyroid stimulating hormone (TSH)	0.89	0.4–4.50 mU/L
Free T4	16.1	9.0–26 pmol/L
Prolactin	4.9	2.7–16.9 mg/L
Cortisol (random)	397	nmol/L
Testosterone	<0.7	6.8–20 nmol/L
Estradiol	<18	55–165 pmol/L
Follicle stimulating hormone (FSH)	0.5	1.6–11 U/L
Luteinizing hormone (LH)	<0.1	0.8–6.1 U/L
Hemoglobin	117	140–175 g/L
